# Ginsenoside Rh4 Suppressed Metastasis of Lung Adenocarcinoma via Inhibiting JAK2/STAT3 Signaling

**DOI:** 10.3390/ijms23042018

**Published:** 2022-02-11

**Authors:** Yan Zhang, Pei Ma, Zhiguang Duan, Yannan Liu, Yu Mi, Daidi Fan

**Affiliations:** 1 Shaanxi Key Laboratory of Degradable Biomedical Materials, Shaanxi R&D Center of Biomaterials and Fermentation Engineering, School of Chemical Engineering, Northwest University, Xi’an 710069, China; zh_yan99@163.com (Y.Z.); mapei@nwu.edu.cn (P.M.); duanzhiguang@nwu.edu.cn (Z.D.); liuyannan@nwu.edu.cn (Y.L.); 2 Biotech & Biomed Research Institute, Northwest University, Xi’an 710069, China

**Keywords:** ginsenoside Rh4, lung adenocarcinoma, metastasis, cell cycle, JAK/STAT signaling

## Abstract

Lung adenocarcinoma (LAC) is a common lung cancer with a high malignancy that urgently needs to be treated with effective drugs. Ginsenoside Rh4 exhibits outstanding antitumor activities. However, few studies reported its effects on growth, metastasis and molecular mechanisms in LAC. Here, Rh4 is certified to show a strong anti-LAC efficiency in vitro and in vivo. Results of flow cytometry and Western blot are obtained to exhibited that Rh4 markedly restrained cellular proliferation and colony formation by arresting the cell cycle in the G1 phase. Results from a wound healing assay and transwell assays demonstrated that Rh4 is active in the antimigration and anti-invasion of LAC. The analysis of Western blot, immunofluorescence and RT-qPCR confirmed that Rh4 reverses the epithelial–mesenchymal transition (EMT) through upregulating the gene expression of E-cadherin and downregulating that of snail, N-cadherin and vimentin. In vivo results from immunohistochemistry show consistent trends with cellular studies. Furthermore, Rh4 suppresses the Janus kinases2/signal transducer and activator of the transcription3 (JAK2/STAT3) signaling pathway stimulated by TGF-β1. Silencing the STAT3 signal or co-treating with AG490 both enhanced the EMT attenuation caused by Rh4, which revealed that Rh4 suppressed EMT via inhibiting the JAK2/STAT3 signaling pathway. These findings explore the capacity and mechanism of Rh4 on the antimetastasis of LAC, providing evidence for Rh4 to LAC therapy.

## 1. Introduction

Lung cancer is one of the most intractable malignant tumors in the world due to its highest mortality. According to the estimates from *CA: A Cancer Journal for Clinicians*, 2.2 million cases and 1.8 million deaths caused by lung cancer occurred in the year of 2020 [1]. Among them, lung adenocarcinoma (LAC) nearly accounted for a proportion of 50% [2]. The high mortality and poor prognosis of cancer have been the most troubling problems in clinical practice over the years. In fact, most primary tumors can be cured by surgery and chemotherapy with a quite good prognosis if diagnosed early. However, it is unfortunate that more than half of lung cancer patients are at the metastatic stage, having only a 5% five-year survival rate [3]. The most important cause of a poor prognosis is the migration and invasion of tumor cells, and these patients have a reduced response to therapeutics once distant metastasis occurs [4]. Therefore, the identification of new drugs and strategies, as well as the underlying mechanisms, are pressing issues requiring solving to improve the current therapy situation and reduce patient mortality.

Metastasis is a continuous and cyclical procedure of tumor cells, which consists of growth, migration and invasion. Thereinto, the epithelial–mesenchymal transition (EMT) is regarded as an initial procedure in tumor metastasis, in which epithelial cells turn into mesenchymal phenotypes, accompanying a cell connection decrease and cell motility increase [5]. It was found that there are EMT-related genes expressed at the edge of part-infiltrated tumors, which implies that the EMT might be a necessary process for tumor metastasis [6]. Bai et al. confirmed that the inhibition of the EMT of breast cancer cells could effectively prevent cellular proliferation and lung metastasis [7]. A recent study pointed out that emodin intake reduced metastatic recurrence in lungs after the surgery of breast cancer by suppressing the EMT in the primary tumors [8], suggesting the potential therapeutic effect of natural drugs on tumor metastasis [9].

In recent years, the natural drug, ginseng, has been experiencing increasing attraction due to its great efficacy and low number of side effects. Ginsenosides, regarded as important active ingredients of ginseng, have excellent pharmacological activities, including antitumor [10,11,12], antibacterial [13], anti-inflammatory [14] and antiobesity effects [15], etc. Nowadays, more and more researchers have turned to their effects on the antiproliferation and antimetastasis of cancer. For instance, ginsenoside Rh2 mediated the apoptosis of A549 cells by targeting the JNK pathway [16] and ginsenoside Rd was reported to attenuate the lung metastasis of breast cancer [17]. In a variety of saponin monomers, ginsenoside Rh4, a tetracyclic triterpenoid saponin, is derived from *Panax notoginseng* root and it is composed of a triterpenoid aglycon and a glycoside [18]. Several studies have proved that Rh4 induced the apoptosis and autophagy of colorectal cancer [19] and resisted the growth of esophageal cancer [20]. However, the efficacy of ginsenoside Rh4 on LAC and the relative mechanism have rarely been studied.

Transforming growth factor-β1 (TGF-β1) regulates several cellular processes, such as proliferation, differentiation and migration, as a typical cytokine. It is often applied to stimulate the EMT of tumor epithelial cells by regulating relative protein expressions [21]. It has been revealed that ginsenoside Rk1 and Rg5 suppressed the EMT process of lung cancer induced by TGF-β1 [22], and ginsenoside Rb2 could enhance E-cadherin, but weaken expressions of N-cadherin, vimentin and snail by mediating the TGF-β1/Smad signaling pathway [23].

The Janus kinases2/signal transducer and activator of the transcription3 (JAK2/STAT3) signaling pathway, a downstream pathway regulated by TGF-β1, is abnormally activated in many kinds of cancers. It provides a great contribution to tumor growth, transformation, the EMT and metastasis [24,25]. Clinical reports have indicated that the overexpression of STAT3 conduced to the progression of breast cancer [26]. Blocking STAT3 inhibited the growth, invasion and migration of colorectal carcinoma [27], hepatic carcinoma [28] and LAC cells [29]. Recent studies have proved that ginsenoside caused an inhibition effect to occur on the STAT3 signal [30,31] and ginsenoside Rh1 was explored to prevent the migration and invasion of MBA-MD-231 cells through blocking STAT3/NF-κB signaling [32]. Hence, targeting the JAK2/STAT3 signaling pathway can be a potential strategy to restrain LAC metastasis.

In this paper, according to the above considerations, the anti-LAC bioactivity of ginsenoside Rh4 and its effect on the restriction of LAC metastasis are explored through an MTT assay, colony formation, cell cycle assay, wound healing assay, transwell assays and the analysis of the protein content. The findings suggested that Rh4 visibly reduced the growth of LAC cells by blocking the cell cycle progress in the G1 phase in vitro and in vivo, and suppressed the EMT via inhibiting the JAK2/STAT3 signaling pathway, providing credible evidence for developing Rh4 as a supplementary drug for LAC.

## 2. Results

### 2.1. Rh4 Inhibited LAC Cell Growth In Vitro and In Vivo

**Figure 1:**
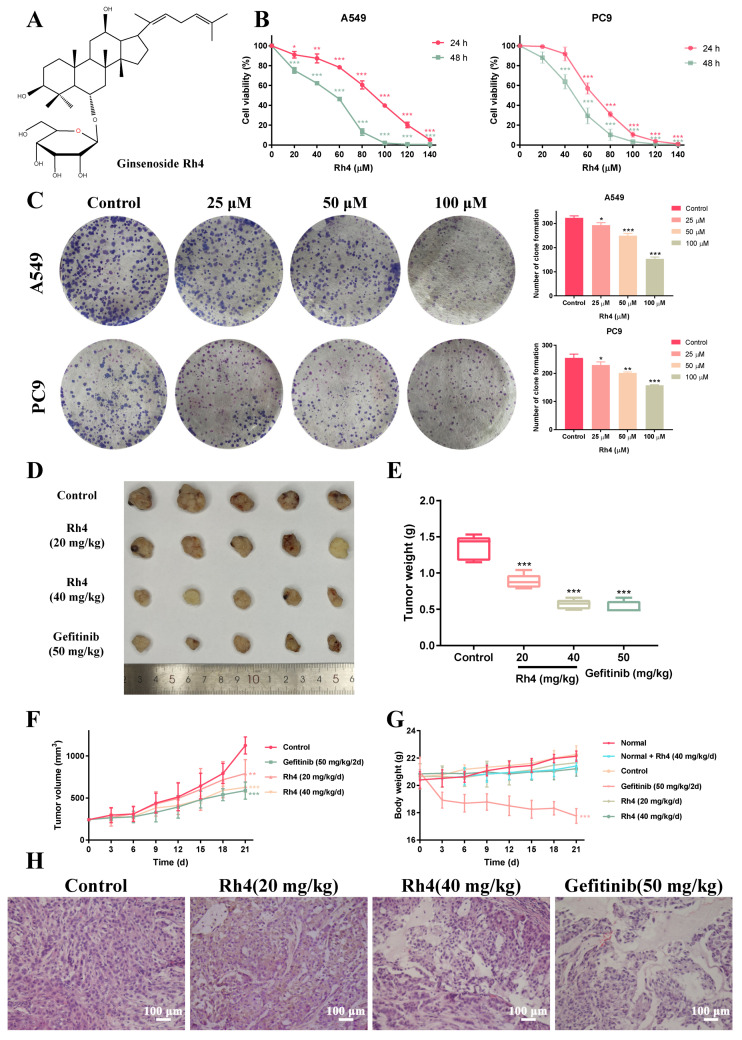
Ginsenoside Rh4 inhibited the growth of LAC cells in vitro and in vivo. (**A**) The chemical structural formula of ginsenoside Rh4. (**B**) MTT assay. Cell viability of human LAC cells A549 and PC9 was measured after treating with Rh4 (0, 20, 40, 60, 80, 100, 120, 140 μM) for 24 h or 48 h. Rh4 significantly inhibited the proliferation of A549 and PC9 cell in a dose- and time-dependent way. (**C**) Colony formation assay. Rh4 (0, 25, 50, 100 μM) reduced the colony formation of A549 and PC9. (**D**) Representative pictures of A549-xenograft tumors of the control group, Rh4-treated groups and gefitinib-treated group. (**E**) Tumor weight of xenograft mice in each group on the 21st day. (**F**) Tumor volume and (**G**) body weight were measured every 3 days. (**H**) *H*&*E* staining of solid tumors in tumor-bearing groups. Rh4 damaged the tight structure of solid tumor. There were 5 mice in each group. Scale bars = 100 µm. Data are presented as means ± SD of three independent experiments, * *p* < 0.05, ** *p* < 0.01 and *** *p* < 0.001 compared with the control.

In order to explore the antitumor activity of Rh4 on LAC cell lines A549 and PC9, the cell viability was determined through an MTT assay. The results revealed that Rh4 notably reduced the viability of A549 and PC9, and when the Rh4 concentration reached 100 µM, the survival rate went down below 20% (Figure 1B). According to estimates from the Probit model, the half maximal inhibitory concentration (IC_50_) was 88.75 µM for A549 and 66.32 µM for PC9 after 24 h. Compared with the control, Rh4 markedly reduced the size and number of colony formations of A549 and PC9 (Figure 1C), showing that ginsenoside Rh4 obviously suppressed the proliferation of LAC cells in vitro.

To provide more evidence to the negative effect of Rh4 on tumor progression, LAC xenograft models were created with A549 cells. The tumor size and its weight were both decreased by Rh4 and gefitinib separately (Figure 1D,E). Volumes of tumors were measured every 3 days during the experimental period and records demonstrated that Rh4 and gefitinib visibly inhibited solid tumor growth (Figure 1F). The inhibition rates of Rh4 (20 mg/kg/d and 40 mg/kg/d) and gefitinib (50 mg/kg/2 d) on tumor volume were, respectively, 29.75%, 44.34% and 47.74%. After 21 days of intraperitoneal injection, the average body weight of mice with gefitinib injection was 17.76 ± 0.48 g, which was an outstanding decrease compared to the normal group (22.14 ± 0.34 g) (*** *p* < 0.001), whereas the weight of mice in the Rh4 injection groups were 21.42 ± 0.40 g in the normal +40 mg/kg Rh4 group, 21.66 ± 0.51 g in the tumor-bearing mice with 20 mg/kg Rh4 group and 21.22 ± 0.50 g in 40 mg/kg Rh4 group, respectively, which were near to the normal group with no significance (Figure 1G, *p* > 0.05). Subsidiarily, results of the hematoxylin and eosin (*H*&*E*) staining assay showed that Rh4 injured the structure of the solid tumor, where the cells were arranged loosely and stained lightly, which were not similar to the control (Figure 1H). Consequently, ginsenoside Rh4 could effectively prevent the growth of LAC cells in vivo.

### 2.2. Low Toxicity Was Induced by Rh4 in Nude Mice

**Figure 2:**
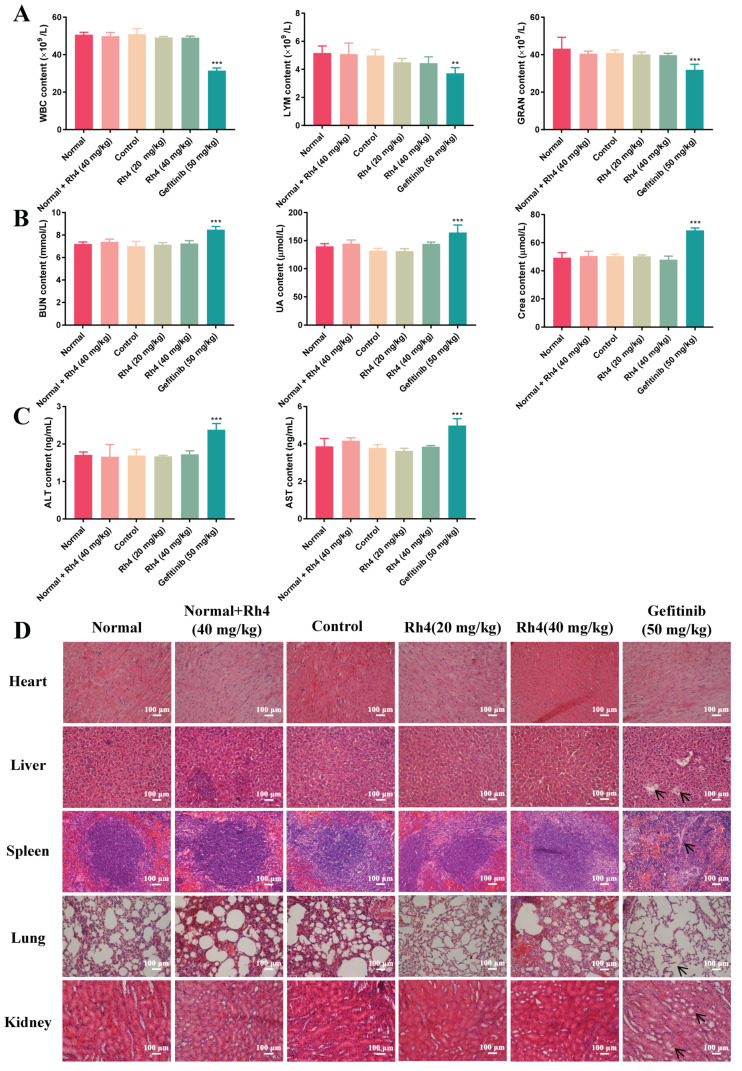
Ginsenoside Rh4 contributed to lower number of side effect of major organs and immune function in xenograft nude mice than gefitinib. (**A**) Count of white blood cells (WBC), lymphocytes (LYM) and granulocytes (GRAN) in peripheral blood of mice in each group were measured using automatic hematology analyzer. The levels of (**B**) renal function parameters (BUN, UA and Crea) and (**C**) liver function parameters (ALT and AST) in the serum from each group. (**D**) *H*&*E* staining of major organs in each group including the heart, liver, spleen, lungs and kidneys. Rh4 caused few damages to major organs of nude mice. There were 5 mice in each group. Scale bars = 100 µm. Data are presented as means ± SD of three independent experiments, ** *p* < 0.01 and *** *p* < 0.001 compared with the normal.

In order to further evaluate the toxicity of Rh4 after preliminarily proving that the side effects of Rh4 on nude mice was much lower than the clinical drug gefitinib, the number of immune cells in experimental groups was measured (Figure 2A). There was not a visible difference in the count of WBC, LYM and GRAN between groups with the normal and Rh4 injection (*p* > 0.05). However, immune cells were markedly reduced by gefitinib (*** *p* < 0.001). It could be confirmed that gefitinib damaged the immune system in nude mice, while Rh4 was considered to be low toxicity. The levels of hepatic and renal function indicators were tested using ELISA kits. The difference from the abnormal increase (*** *p* < 0.001) in the gefitinib group was that Rh4 did not cause accumulations of ALT and AST, two common liver function parameters, or BUN, UA and Crea, three common renal function parameters (*p* > 0.05), which indicated minimal side effects of Rh4 on the liver and kidneys of nude mice (Figure 2B,C). Furthermore, the *H*&*E* staining exhibited that there were hardly any pathological changes of vital organs in the normal group and Rh4 treatment groups. Otherwise, liver and renal vacuolation, alveolar septum widening and a spleen structure disorder occurred in the gefitinib group (Figure 2D). These results illustrated that Rh4 induced a low toxicity of vital organ functions and the immune system in vivo.

### 2.3. Rh4 Induced Cell Cycle Arrest in G1 Phase

**Figure 3:**
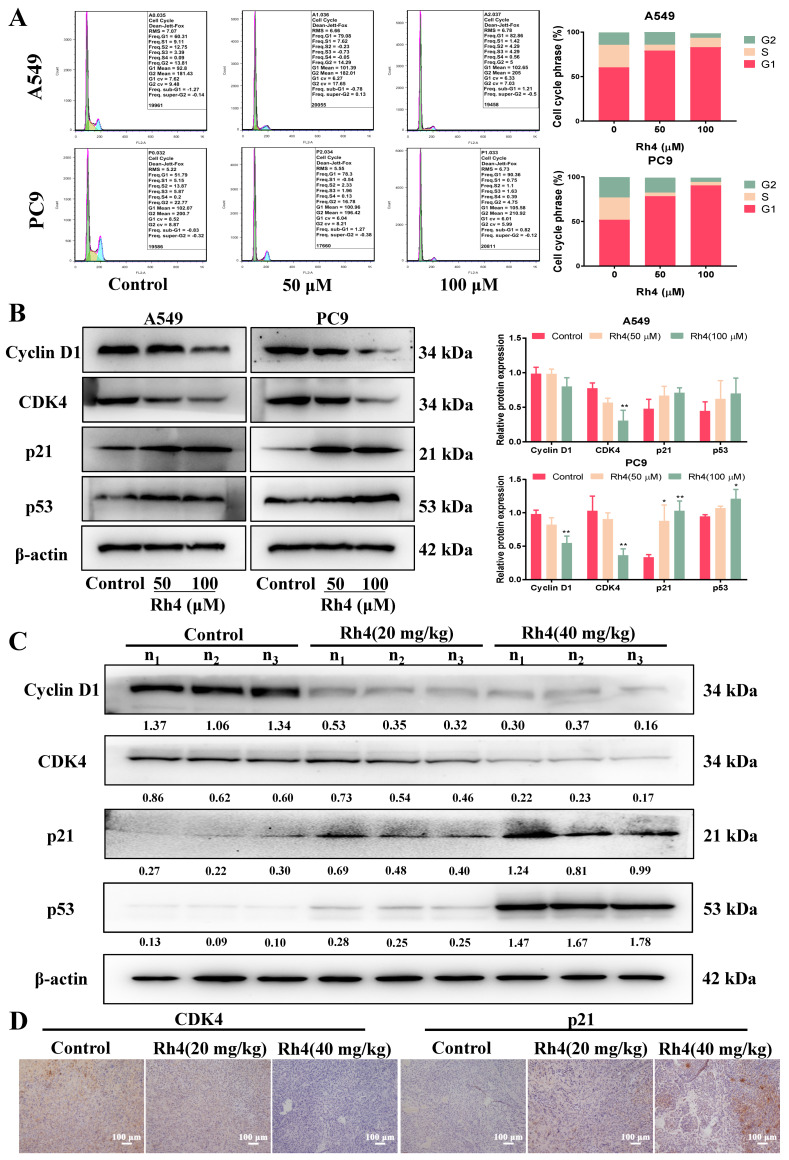
Ginsenoside Rh4 triggered cell cycle of LAC cells arrest in G1 phase in vivo and in vitro. (**A**) Flow cytometry. Rh4 increased the proportion of cells in the G1 phase but decreased cells in S phase and G2 phase. (**B**,**C**) Western blot analysis. Expressions of proteins related to G1 phase in vivo and in vitro (CyclinD1, CDK4, p21 and p53) were measured and Rh4 enhanced p21 and p53 and reduced CyclinD1 and CDK4 in LAC cells (**B**) and tumor tissues (**C**) in a dose-dependent manner. (**D**) Immunohistochemistry staining. Rh4 reduced the expression of CDK4 but upregulated p21 in tumor tissues. β-actin was applied to be an endogenous reference. n_1_, n_2_ and n_3_ represented three different nude mice. There were 5 mice in each group. Scale bars = 100 µm. Data are presented as means ± SD of three independent experiments, * *p* < 0.05 and ** *p* < 0.01 compared with the control.

To certify whether Rh4 induced cell cycle redistribution in LAC, flow cytometry was firstly performed. In contrast with the control, the proportion of A549 cells in the G1 phase increased from 60.31% to 82.86% under the 100 μM Rh4 treatment, and the proportion of PC9 cells in the G1 phase increased from 51.79% to 90.36%. Cells in both the S and G2 phases decreased in A549 and PC9 (Figure 3A). In addition, Western blot was performed to analyze the level of proteins associated with the G1 phase, and results presented that Rh4 conspicuously downregulated Cyclin D1 and CDK4 but upregulated p21 and p53 dose-dependently in LAC cells (Figure 3B, * *p* < 0.05).

The protein expression of the G1 phase was detected in vivo through Western blot and immunohistochemistry. Consequences showed that Rh4 notably lessened Cyclin D1 and CDK4, but raised p21 and p53, which were consistent with cellular experiments (Figure 3C). The same variation of CDK4 and p21 was observed in the immunohistochemistry assay (Figure 3D). All the above results attested that ginsenoside Rh4 induced cell death of LAC by means of cell cycle arrest in the G1 phase in vitro and in vivo. 

### 2.4. Rh4 Restrained Migration and Invasion of A549 and PC9

**Figure 4:**
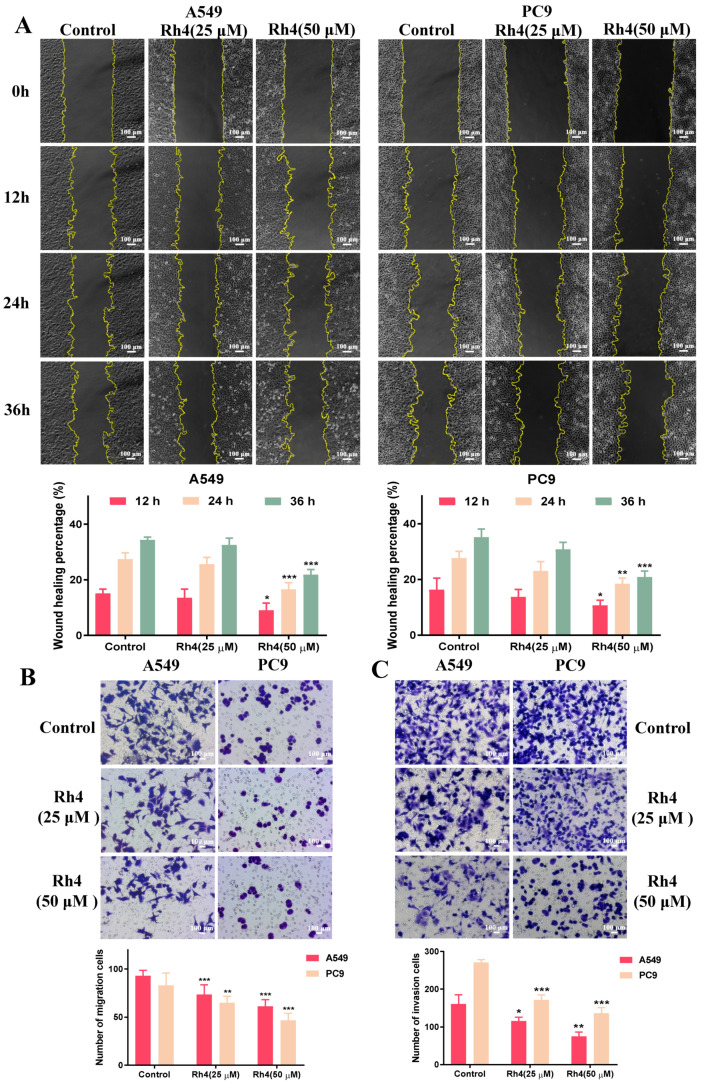
Ginsenoside Rh4 significantly suppressed migration and invasion of LAC cells. (**A**) Wound healing assay. Rh4 reduced the wound healing percentage of A549 and PC9. (**B**,**C**) Transwell assays. Number of migration cells (**B**) and invasion cells (**C**) were reduced by Rh4. Scale bars = 100 µm. Data are presented as means ± SD of at least three independent experiments, * *p* < 0.05, ** *p* < 0.01 and *** *p* < 0.001 compared with the control.

To explore the antimigration and anti-invasion effects of Rh4 on LAC cells after demonstrating the antigrowth effect of Rh4, the wound healing assay and transwell assays were designed. The findings confirmed that Rh4 markedly suppressed the capacity of the migration and invasion of A549 and PC9. After incubation with Rh4 of 25 μM and 50 μM for 12 h, 24 h or 36 h, the wound healing percentages were evidently decreased, which suggested that Rh4 inhibited the migration ability of LAC cells (Figure 4A). The transwell assay for migration proved that the Rh4 (50 μM) treatment drastically reduced the count of cells migrating to the lower compartment from 93.00 ± 5.27 to 61.25 ± 6.44 for A549 and from 83.13 ± 11.93 to 46.88 ± 6.64 for PC9 (Figure 4B, * *p* < 0.05,). Subsequently, the cell invasion was investigated through a transwell assay, and it was discovered that Rh4 markedly inhibited the count of cells invading down to the lower compartment after matrix gel dissolution (Figure 4C, ** *p* < 0.01).

### 2.5. Rh4 Reversed EMT Induced by TGF-β1 In Vitro and In Vivo

**Figure 5:**
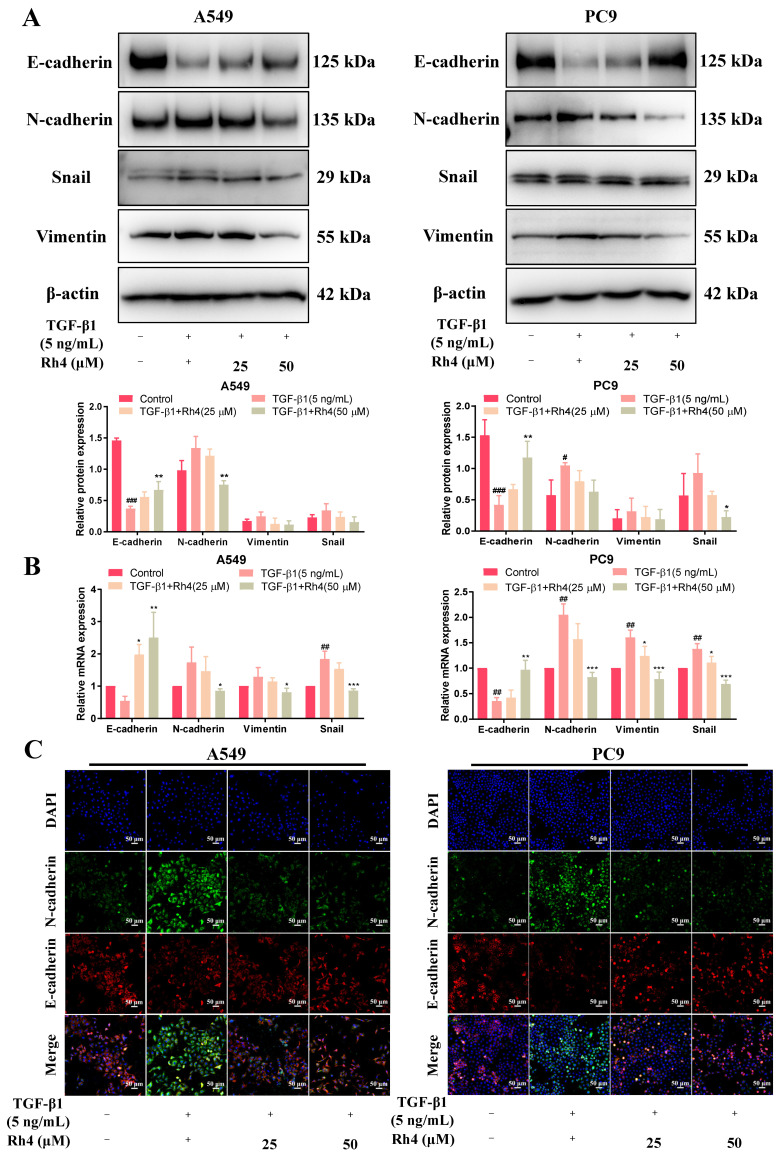
Ginsenoside Rh4 reversed TGF-β1-induced EMT of LAC cells (A549 and PC9). (**A**) Western blot analysis. TGF-β1 stimulated the expression of N-cadherin, vimentin and snail, while they were averted by Rh4; the expression of E-cadherin was opposite. (**B**) RT-qPCR assay was carried out to detect the relative content of EMT-related mRNA in LAC cells (A549 and PC9). (**C**) Immunofluorescence assay. Rh4 reversed the downregulation of E-cadherin and the upregulation of N-cadherin induced by TGF-β1. β-actin was used as an endogenous reference. Scale bars = 50 µm. Data are presented from at least three independent experiments, ^#^ *p* < 0.05, ^##^ *p* < 0.01 and ^###^*p* < 0.001 compared with the control, * *p* < 0.05, ** *p* < 0.01 and *** *p* < 0.001 compared with the TGF-β1 (5 ng/mL) group.

Having confirmed that Rh4 could reduce the migration behavior of LAC cells, the effect of Rh4 on the EMT process was explored. Briefly, A549 and PC9 cells were firstly stimulated to undergo mesenchymal transformation using TGF-β1 (5 ng/mL) and then treated with different doses of Rh4 (0, 25, 50 μM) for 24 h. The total cellular protein was extracted for protein expression determination. An analysis exhibited that the E-cadherin expression was visibly downregulated both in A549 and PC9 cells after inducing by TGF-β1, but was increased by Rh4. Oppositely, expressions of N-cadherin, vimentin and snail were markedly enhanced by TGF-β1, while being weakened after treating with Rh4 (Figure 5A). In addition, results of RT-qPCR displayed the consistent varying trends for mRNA expression levels. In detail, stimulated by TGF-β1, the mRNA expressions of mesenchymal biomarkers were promoted greatly, while the mRNA expression of E-cadherin, an epithelial biomarker, was attenuated. However, Rh4 reduced the mRNA levels of mesenchymal biomarkers and increased that of E-cadherin (Figure 5B). Changes in the protein content of A549 and PC9 were evaluated more distinctly by performing an immunofluorescence assay, and it could be observed that Rh4 restored the level of E-cadherin that was reduced by TGF-β1 (Figure 5C). Collectively, it was implied that ginsenoside Rh4 reversed the EMT process stimulated by TGF-β1 by interfering with the expression of N-cadherin, E-cadherin and other biomarkers. 

**Figure 6:**
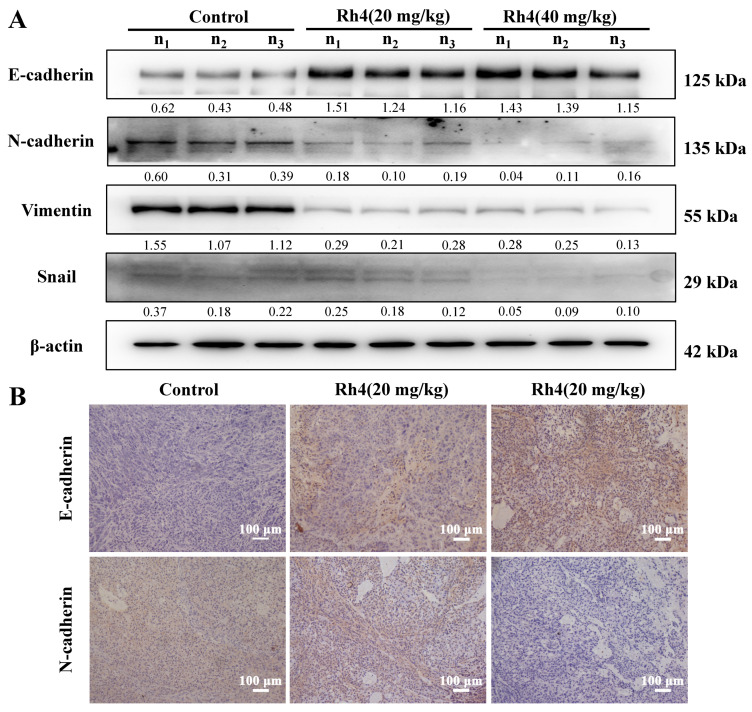
Ginsenoside Rh4 refrained the EMT procedure of LAC in vivo. (**A**) Western bolt analysis. Rh4 promoted the expression of E-cadherin, while inhibiting the expression of N-cadherin, vimentin and snail in tumor tissues via Western blot analysis. (**B**) Immunohistochemistry was carried out to evaluated the variations of E-cadherin and N-cadherin. β-actin was used as an endogenous reference. n1, n2 and n3 represented three different nude mice. Scale bars = 100 µm. Data are presented from three independent experiments.

Western blot and immunohistochemical staining were also carried out to detect expressions of EMT-related proteins in vivo and the varying trends of these proteins were consistent with that in vitro (Figure 6A,B). 

### 2.6. JAK2/STAT3 Was Involved in EMT Suppression by Rh4

**Figure 7:**
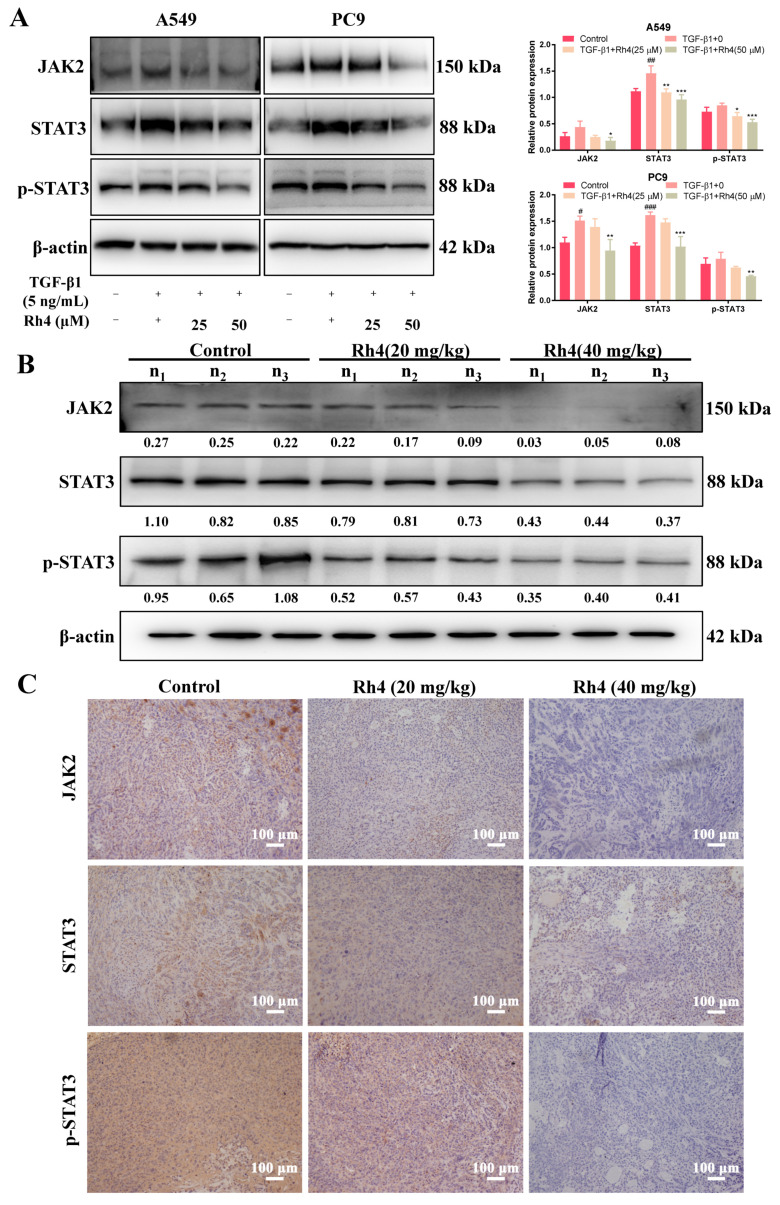

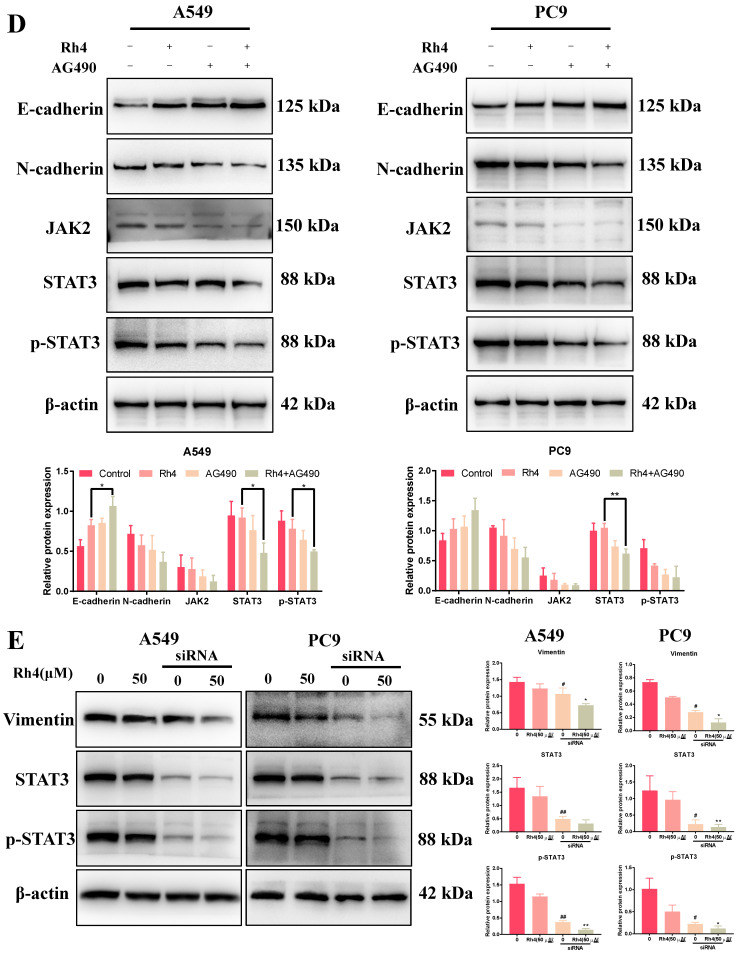
Ginsenoside Rh4 attenuated JAK2/STAT3 signaling pathway of LAC in vivo and in vitro. (**A**) Western blot analysis. Rh4 inhibited the upregulation of JAK2, STAT3 and p-STAT3 by TGF-β1 in LAC cells from Western blot analysis. (**B**,**C**) Rh4 decreased the expression of JAK2, STAT3 and p-STAT3 in vivo, which were detected by Western blot (**B**) and immunohistochemistry staining (**C**). (**D**) Western blot analysis. The effect of Rh4 combined with AG490 on expressions of EMT biomarkers and JAK2/STAT3 proteins in LAC cells (A549 and PC9). (**E**) Western blot analysis. The effect of Rh4 combined with STAT3 siRNA expressions of EMT biomarker in LAC cells (A549 and PC9). β-actin was used as an endogenous reference. n_1_, n_2_ and n_3_ represent three different mice in each group. There were 5 mice in each group. Scale bars = 100 µm. Data are presented as mean ± SD of three independent experiments, ^#^ *p* < 0.05, ^##^ *p* < 0.01 and ^###^ *p* < 0.001compared with the negative control, * *p* < 0.05, ** *p* < 0.01 and *** *p* < 0.001 compared with the TGF-β1 (5 ng/mL) group (**A**) or Rh4 group (**E**).

In order to investigate how Rh4 attenuated the EMT process of LAC, JAK2/STAT3 signaling occurred as a pathway that regulated by TGF-β1. The STAT3 signal is closely associated with the EMT in many kinds of cancers, including breast cancer [33], gastric cancer [34], colorectal cancer [35] as well as LAC [36]. Recent evidence showed that the activated JAK/STAT3 pathway was a momentous signal for TGF-β1-induced EMT in lung cancer [37]. The analysis in this study also proved that, compared to the control, expressions of JAK2 and STAT3 were evidently increased by TGF-β1, and STAT3 phosphorylation was, likewise, strengthened. Nevertheless, Rh4 blocked the enhancement of JAK2 and STAT3, as well as STAT3 phosphorylation (Figure 7A). In vivo, the expression of JAK2, STAT3 and p-STAT3 was also inhibited via the analysis of Western blot and immunohistochemistry (Figure 7B,C).

Moreover, the specific inhibitor AG490 was used to probe the key role of JAK2/STAT3 signaling in the EMT of LAC cells. In total, 50 μM AG490 was add into A549 and PC9 cells for pretreatment for 3 h, and then cells were cocultured with or without Rh4 (50 μM). Compared with the negative control (without AG490), the expressions of JAK2, STAT3 and p-STAT3 in the AG490 group were suppressed, and Rh4 (50 μM) further downregulated the level of these JAK2/STAT3-related proteins. Moreover, coincubating with AG490 and Rh4 enhanced the E-cadherin expression and reduced N-cadherin significantly (Figure 7D). The siRNA transfection assay of STAT3 displayed that the STAT3 reduction and inactivation restrained vimentin expression (Figure 7E). Consequently, these results suggested that ginsenoside Rh4 suppressed the EMT of LAC cells via mediating the JAK2/STAT3 signaling pathway.

## 3. Discussion

**Figure 8:**
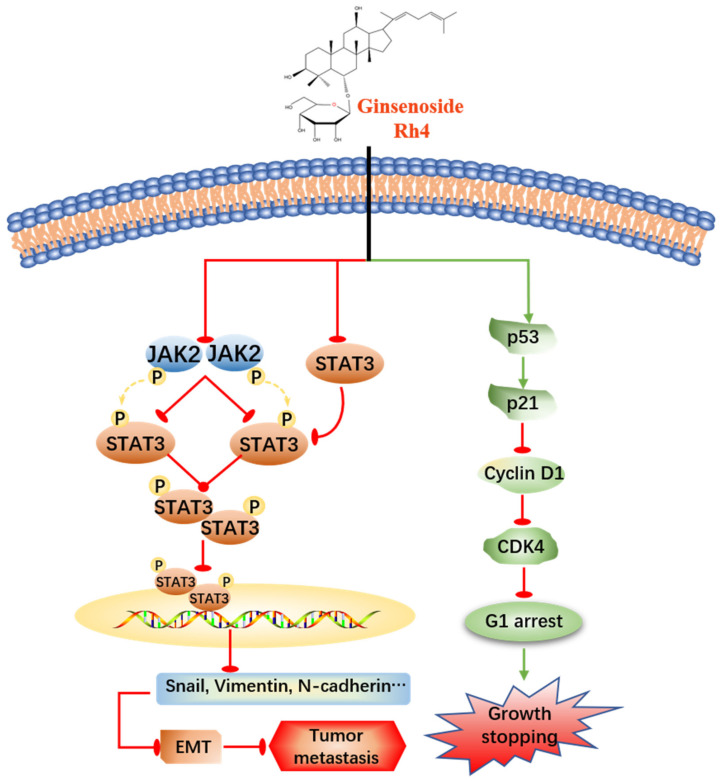
Proposed molecular mechanism of the anti-LAC activity of Rh4. Rh4 inhibited the proliferation of LAC cells via triggering cell cycle arrest in G1 phase and suppressing the EMT procedure by blocking JAK2/STAT3 signaling pathway.

The natural drug ginsenoside Rh4 was proved to have anti-inflammatory [38], antidiabetic [39], antianemia [40] as well as anticancer properties. Our studies published previously testified that ginsenoside Rh4 motivated the apoptosis of colorectal cancer and breast cancer [19,41]. Lung cancer is the leading cause of cancer-related deaths worldwide. There are statistics presenting that 90% deaths of cancer patients are caused by tumor metastasis [42]. Metastasis is closely related to a complex series of biological procedures consisting of the EMT, invasion, migration, MET (mesenchymal–epithelial transition) and colony formation, etc., which was called the invasion–metastasis cascade [43]. In this study, ginsenoside Rh4 was demonstrated in vitro and in vivo to effectively restrain proliferation by triggering cell cycle arrest in the G1 phase and markedly suppressing the EMT process by blocking the activation of JAK2/STAT3 signaling, which preliminarily explored the antimetastasis activities of Rh4 (Figure 8).

In vitro, the cell viability and colony formation were obviously decreased by Rh4, and the survival ratios were both below 10% when the dose was 100 μM after 48 h of treatment. In vivo, the volume inhibition of solid tumors with 20 mg/kg and 40 mg/kg Rh4 intraperitoneal injection were 29.74% and 44.34%, respectively, and the tumor constructions were obviously damaged, which indicated the excellent efficacy of Rh4. At the same time, Rh4 caused no weight loss of tumor-bearing nude mice, which was different from gefitinib. Moreover, the measurement of serum biochemical parameters and *H*&*E* staining exhibited that ginsenoside Rh4 had no negative effect on the normal structure and function of the heart, lungs, liver, spleen or kidneys, and the blood test showed that Rh4 caused no reduction in the number of immune cells, pointing out that Rh4 induces little toxicity to the body during the LAC treatment. Hence, ginsenoside Rh4 had quite a strong inhibition effect on LAC in vivo and in vitro and induced a low toxicity to nude mice, which suggested it may be developed to be available as an anti-LAC drug.

The cell cycle, covering from the end of one division to the start of another division of the proliferating cell, is regarded as a vital pathway to regulate cell growth. Arresting the cell cycle is reported to be an efficient method to inhibit tumor growth [44,45]. There is an independent system controlling the cell cycle and the core of the system is cyclin-dependent kinases (CDKs), whose expression level varies during cell proliferation [46]. Moreover, transcription factor p53 is a crucial factor for repairing DNA damage by upregulating the level of p21 and blocking the cell cycle in the G1 phase [47]. Therefore, triggering a cell cycle arrest through affecting the level of related proteins has been considered as a potential approach to tumor treatment. Herein, the proportion of cells in the G1 phase visibly increased after Rh4 treatment, both in A549 and PC9, via the measurement of flow cytometry, along with a decrease in the proportion in the S and G2 phases. Consistently, the Western blot analysis showed that Rh4 increased p53 and p21, while reducing CDK4 and cyclin D1, both in vitro and in vivo, indicating that Rh4 stopped the growth of LAC by motivating a cell cycle arrest in the G1 phase.

The EMT is an initial and vital procedure of tumor metastasis, which is highly conserved in a variety of cells [5]. Under the activities of several factors, such as the tumor microenvironment (TME), a cluster of tumor epithelial cells loses its tight junction and mutual adhesion among cells and transforms into mesenchymal cells with a long spindle shape and related characteristics, obtaining the properties to invade and migrate to other issues [48,49]. It has been shown that the continuous EMT process is closely relevant to tumor metastasis [50], accompanied with a series of varying proteins. Among these biomarkers, epithelial cadherin (E-cadherin), which is responsible for maintaining the tight connections between cells, is reduced in the EMT. Neuro cadherin (N-cadherin) and vimentin are the signature proteins of mesenchymal cells, and vimentin is an important component protein of cytoskeleton, with a high expression in mesenchymal cells. In addition, the increase in the transcription factor snail inhibited the expression of E-cadherin. By establishing caudal vein metastasis models of tumor cells with EMT-specific gene overexpression (Twist), Yang et al. found that promoting the EMT of tumor cells could increase their capacity to participate in blood circulation [51]. In our study, the wound healing assay and transwell assays proved that coincubating with ginsenoside Rh4 significantly attenuated the migration and invasion of LAC cells. It was obtained from the Western blot analysis and immunohistochemistry that Rh4 weakened the expression of N-cadherin, while it increased E-cadherin in vivo and in vitro, suggesting that the EMT of LAC cells was visibly suppressed.

TGF-β1 is a cytokine that regulates cell growth and differentiation, which promotes the EMT of tumor cells and cancer development [52]. The level of TGF-β1 in tumor cells was significantly higher than that in epithelial tissues [53,54]. In our study, A549 and PC9 were first stimulated by TGF-β1 for mesenchymal transformation and E-cadherin was downregulated, but N-cadherin, vimentin and snail were augmented. Afterwards, the Rh4 treatment reversed the expression changes of these proteins, implying that Rh4 conspicuously restrained the EMT of LAC cells. These results were further testified at the mRNA level through the RT-qPCR experiment. Consistent with our results, Seong et al. reported that BIX02189, the MEK5 pathway inhibitor, reduced the migration of LAC and suppressed the EMT procedure induced by TGF-β1 through regulating the expression of E-cadherin and N-cadherin [21].

Previous research explored that TGF-β1 provoked the EMT of LAC via the JAK2/STAT3 signaling pathway [36], which was reported to be greatly important in many cellular processes such as proliferation, apoptosis, invasion and metastasis. Moreover, JAK2/STAT3 is also involved in tumor genesis and development [55]. Many studies demonstrated that targeting the JAK2/STAT3 signal is an effective therapy for lung cancer metastasis. Wu et al. analyzed a large number of papers and summarized that poor prognosis in lung cancer patients was associated with the overexpression of STAT3, who experienced a later clinical tumor stage and a lower 3-year overall survival rate [56]. Chuang et al. determined that the reduction in STAT3 inhibited LAC metastasis [57]. Similarly, the JAK2/STAT3 signaling was motivated by TGF-β1, but was prevented with the Rh4 treatment. From the analysis of cellular protein and tumor tissue protein by Western Blot and immunohistochemistry, it could be illustrated that Rh4 dramatically inhibited the expression of JAK2, STAT3 and p-STAT3, blocking the EMT procedure in LAC cells. The specific inhibitor of JAK2/STAT3, AG490, could also enhance the adverse effect on the EMT and silencing STAT3 expression by the STAT3-siRNA transfection experiment displaying a consistent conclusion. In a word, our discoveries demonstrated that ginsenoside Rh4 suppressed the metastasis of LAC via inhibiting JAK2/STAT3 signaling.

## 4. Materials and Methods

### 4.1. Materials and Chemicals

Ginsenoside Rh4 (Figure 1A, purity ≥ 99%) was purchased from Puruifa Technology Development Co., Ltd. (Chengdu, China). Roswell Park Memorial Institute-1640 (RPMI-1640) and fetal bovine serum (FBS) were obtained from Gibco (Thermo Fisher Scientific). Penicillin, streptomycin and methylthiazolyldiphenyl tetrazolium bromide (MTT) were procured from Beijing Solarbio Science & Technology Co., Ltd. (Beijing, China). Gefitinib powders and dimethyl sulfoxide (DMSO) were obtained from Aladdin Biotechnology (Shanghai, China). TGF-β1 and the cell cycle kit were purchased, respectively, from PeproTech (East Windsor, NJ, USA) and KeyGEN BioTECH (Nanjing, China). JAK2/STAT3 pathway inhibitor AG490 was obtained from MedChemExpress (Monmouth Junction, NJ, USA). For Western Blot analysis, primary antibodies against Cyclin D1, CDK4, p21, p53, E-cadherin, N-cadherin and vimentin were purchased from Proteintech Group Inc. (Chicago, IL, USA) and antibodies against snail, JAK2, STAT3, p-STAT3 and β-actin were obtained from Abcam Technology (Cambridge, UK). Goat antirabbit IgG and goat antimouse IgG were purchased from Abbkine (Wuhan, China).

### 4.2. Cell Culture

A549 and PC9 were purchased from the American Type Culture Collection (ATCC, Manassas, VA, USA), which were cultured by RPMI-1640 with 10% FBS and 1% penicillin and streptomycin. The conditions of incubator were 37 °C and 5% CO_2_.

### 4.3. MTT Assay

The proliferation ability of A549 and PC9 was measured through MTT assay. In detail, cells (1 × 10^4^ cells per well) were seeded into 96-well plates for 24 h incubation and Rh4 (0, 20, 40, 60, 80, 100, 120, 140 μM) was added to each group. After 24 h or 48 h, 50 μL MTT (5 mg/mL) solution was added into each sample to incubate for 2–4 h. Finally, purple formazan crystals in the wells were completely dissolved with 150 μL DMSO. All absorbance values of samples at 490 nm were detected in the microplate reader (Power Wave XS2, Bio-Tek Instruments Inc., Winooski, VT, USA) and the Probit method was applied for the half maximal inhibitory concentration (IC_50_).

### 4.4. Colony Formation Assay

Cells in 6-well plates (500 cells per well) were first treated with Rh4 (0, 25, 50, 100 μM) for 24 h and then cultured in fresh RPMI-1640 for about 2 weeks until colonies were visible to the naked eyes. Methanol was added to fix colonies for 20 min before Giemsa (Beyotime, Shanghai, China) staining. The quantity of colonies was counted through a microscope (Nikon, Tokyo, Japan).

### 4.5. Human LAC Xenograft Nude Mouse Model

In order to evaluate Rh4 efficiency on LAC in vivo, xenograft nude mouse models were constructed using four-week-old female BALB/C nude mice purchased from GemPharmatech LLC (Jiangsu, China) and their weights were 14 ± 2 g. All these athymic mice were supplied with sterile feeding all throughout the experiment. After adaption for 5 days, some mice had a subcutaneous inoculation performed in the left forelimb armpit using A549 in a concentration of 2 × 10^7^ cells per mouse. When the volume of solid tumor was up to 150 mm^3^, mice were divided into 6 groups (*n* = 5) as follows: normal group (not tumor-bearing), normal + 40 mg/kg/d Rh4 group, control group (tumor-bearing without drug), 50 mg/kg/2 d gefitinib group [23], 20 mg/kg/d and 40 mg/kg/d Rh4 groups. Gefitinib and Rh4 were given by intraperitoneal injection. Every 3 days, mice were weighed and tumor volumes were measured. The tumor volumes were estimated by the equation of volume = (length × width^2^)/2. After 21 days of injections, mice in all groups were euthanized and serum samples were isolated from blood. Solid tumors and crucial organs were separated, weighed, and then kept at −80 °C. The whole period of experiments on mice were finished under the requirements of the Laboratory Animal Act of the People’s Republic of China and approved by the Animal Ethics Committee of Northwest University (NWU-AWC-20210304M).

### 4.6. Cell Cycle Assay by Flow Cytometry

A549 and PC9 were treated by Rh4 at a concentration of 0, 50 and 100 μM. Before staining using RNase A (100 μg/mL) and PI (50 μg/mL) for 30 min in the dark, cells were fixed with 75% ethanol at 4 °C overnight. The fluorescence was detected by flow cytometry (Becton Dickinson, Fullerton, CA, USA). Statistics were analyzed using Flowjo 7.6.1 software.

### 4.7. Wound Healing Assay

Three horizontal lines were drawn in the 6-well plates on the back and LAC cells were seeded in them. When cell confluence had reached above 90% after 24 h cultivation, the monolayer wounds perpendicular to horizontal lines were scratched using a 200 μL pipette tip. Then, PBS was added to lightly wash cells twice prior to incubation in fresh medium with 2% FBS included and with or without Rh4. Next, cells were cultivated at 37 °C. Representative scrape lines of wound healing areas were photographed, respectively, at 12 h, 24 h and 36 h, and wound healing percentages were analyzed with ImageJ software. 

### 4.8. Transwell Assay

For cell migration assays, 8 μm chambers (Merck Millipore, Burlington, MA, USA) containing A549 and PC9 cells (1.0 × 10^4^ per well) and RPMI-1640 (without FBS) were placed into 24-well plates with RPMI-1640 with 20% FBS. Migrated cells were stained with 1% crystal violet (Merck Sigma–Aldrich, St. Louis, MI, USA) after Rh4 treatment for 24 h. Enumeration was carried out under the inverted microscope and mean values of random five fields were calculated. For invasion assays, chambers were first coated with ECM gel (Merck Sigma–Aldrich, St. Louis, MI, USA) before 5.0 × 10^4^ cells were seeded per well. Additionally, the remaining procedure was the same as migration assays. All the experiments were repetitively performed three times.

### 4.9. Immunofluorescence Assay

LAC cells were cultured on the carry sheet glass overnight and coincubated with TGF-β1 (5 ng/mL) for 24 h, which was followed by the treatment of Rh4 (0, 25, 50 μM). After being fixed for 24 h, A549 and PC9 were blocked in 5% BSA for 2 h, followed by incubating with E-cadherin and N-cadherin, respectively, at 4 °C overnight. At last, a second antibody was added to incubate for 3 h. Samples were visualized through an Olympus confocal microscope (Tokyo, Japan).

### 4.10. Western Blot

The total protein was lysed by RIPA (Beyotime, Shanghai, China) with 1% phosphatase inhibitor cocktail (Solarbio, Beijing, China) and 1 mM phenylmethylsulfonyl fluoride (PMSF). The lysates were centrifuged under conditions of 4 °C, 12,000 rpm and 20 min, and concentrations of protein were determined using a BCA kit (Solarbio, Beijing, China). The concentrations of each sample were adjusted to be consistent and then equal amounts of protein (20 μg) were separated by SDS-PAGE. Proteins were transferred to PVDF membranes through Trans-Blot Turbo transfer system (Bio-Rad, Hercules, CA, USA). Then, PVDF membranes were incubated with different primary antibodies at 4 °C overnight and incubated with HRP-linked secondary antibodies. Finally, the enhanced chemiluminescence (ECL) substrate (Merck Millipore, Burlington, MA, USA) with a Gel Image system (Tanon5200, Shanghai, China) was used for proteins detection.

### 4.11. Histopathology and Immunohistochemistry

Tissues of solid tumor and major organs that separated from xenograft nude mice were first fixed by formalin. In the H&E staining, tissues embedded by paraffin were divided into thin 5 µm parts and stained with 10% hematoxylin and 1% eosin. In the immunohistochemistry assay, tumors were stained with CDK4, p21, E-cadherin, N-cadherin, JAK2, STAT3 and p-STAT3. Images were captured under an optical microscope (Nikon, Japan).

### 4.12. Real-Time Quantitative Polymerase Chain Reaction (RT-qPCR)

The total, the RNA of A549 and PC9 cells was extracted by means of TRIzol reagent (Ambion, Austin, TX, USA) and cDNA was synthesized through the reverse-transcription kit (Thermo Fisher, Shanghai, China). The final relative expressions of mRNA were analyzed through a 2^−ΔΔCt^ method. GAPDH was exhibited as the internal control. Primers were as follows: GAPDH: (forward) 5′-GGGGAAGGTGAAGGTCGGAG-3′, (reverse) 5′-TCTCGCTCCTGGAAGATGGTGAT-3′; E-cadherin: (forward) 5′-GGAACTATGAAAAGTGGGCTTG-3′, (reverse) 5′-AAATTGCCAGGCTCAATGAC-3′; N-cadherin: (forward) 5′-GGTGGAGGAGAAGAAGACCAG-3′, (reverse) 5′-GGCATCAGGCTCCACAGT-3′; Vimentin: (forward) 5′-GACGCCATCAACACCGAGTT-3′, (reverse) 5′-CTTTGTCGTTGGTTAGCTGGT-3′; Snail: (forward) 5′-CTTCCAGCAGCCCTACGAC-3′, (reverse) 5′-CGGTGGGGTTGAGGATCT-3′.

### 4.13. Plasmids and siRNA Transfection

A549 and PC9 cells, which were transfected with 100 pM siRNA duplexes using Lipofectamine™2000 (Invitrogen Carlsbad, Carlsbad, CA, USA) for 48 h, were incubated with or without Rh4 (50 μM) for subsequent experiments. The specific siRNA of STAT3 (sense: 5′-CCCGGAAAUUUAACAUUCUTT-3′ and antisense: 5′-AGAAUGUUAAAUUUCCGGGTT-3′) were synthesized by GenePharma (Shanghai, China).

### 4.14. Hemogram Assay and Measurement of Biochemical Parameters

After finishing treatment, peripheral blood samples from orbital venous plexus were diluted with ethylenediaminetetraacetic acid and detected for white blood cells (WBC), lymphocyte (LYM) and granulocyte (GRAN) through automatic hematology analyzer (HC2200, Meiyilinm, China).

On the 21st day, serum samples centrifuged from blood samples of nude mice were analyzed for the renal function parameters UA, BUN and Crea, and the liver function parameters ALT and AST by ELISA kits (Shanghai Enzyme Biotechnology Co., Ltd., Shanghai, China).

### 4.15. Statistical Analysis

Statistical analysis was performed using SPSS version 21.0 software (SPSS Inc., Chicago, IL, USA) and GraphPad Prism 7.0 software. One-way analysis of variance (ANOVA) followed by a Bonferroni multiple comparison was performed. The results were represented as mean ± standard deviation (SD) and from at least triple independent experiments. * *p* < 0.05, ** *p* < 0.01, and *** *p* < 0.001 were regarded as statistically significance.

## 5. Conclusions

Ginsenoside Rh4 decreased the proliferation of LAC in vivo and in vitro in a dose-dependent way by arresting the cell cycle in the G1 phase. Moreover, the migration and invasion of LAC cells were markedly suppressed by Rh4, and the EMT progress was restrained via inhibiting the JAK2/STAT3 signaling pathway. This study revealed the antimetastasis efficiency of Rh4 and the potential molecular mechanism in LAC. Complementally, several basic experiments for a safety evaluation in vivo provided more evidence for the clinical application of Rh4.

## Data Availability

The data presented in this study are available within the article text and figures.
